# Risk factors of posthemorrhagic seizure in spontaneous intracerebral hemorrhage

**DOI:** 10.1007/s10143-025-03229-2

**Published:** 2025-01-23

**Authors:** Apisut Imsamer, Bunpot Sitthinamsuwan, Chottiwat Tansirisithikul, Sarun Nunta-aree

**Affiliations:** 1 Division of Neurosurgery, Department of Surgery, Faculty of Medicine Siriraj Hospital, Mahidol University, 2 Wang Lang Road, Bangkok Noi, Bangkok, 10700 Thailand; 2 Department of Surgery, Vachira Phuket Hospital, Phuket, Thailand

**Keywords:** Seizure, Spontaneous intracerebral hemorrhage, Predictive factor, Risk factor, Hemorrhagic stroke, Early seizure, Late seizure

## Abstract

Seizure is a relatively common neurological consequence after spontaneous intracerebral hemorrhage (SICH). This study aimed to investigate risk factors of early, late, and overall seizures in patients with SICH. Retrospective analysis was performed on all patients with SICH who completed two years of follow-up. The variables collected were obtained from demographic, clinical, radiographic and treatment data, in-hospital complications, and follow-up results. Univariate and multivariate analyzes were used to identify risk factors for post-hemorrhagic stroke seizure. Of 400 SICH patients recruited, 30 (7.5%) and 40 (10%) developed early and late seizures during the 2-year follow-up period, respectively. In the final result of the multivariate analysis, factors associated with the occurrence of the early seizure included lobar location of hematoma (*p* = 0.018), and GCS ≤ 12 on initial clinical presentation (*p* = 0.007). Factors associated with the occurrence of the late seizure included lobar location of hematoma (*p* = 0.001), volume of hematoma greater than 10 ml (*p* = 0.009), and midline shift on initial cranial CT (*p* = 0.036). Risk factors of the overall seizure after SICH included lobar location of hematoma (*p* < 0.001), volume of hematoma greater than 10 ml (*p* < 0.001), and craniotomy with evacuation of hematoma (*p* = 0.007). Furthermore, seizure was also associated with a poor functional outcome 2 years after the onset of SICH. Several factors associated with the appearance of post-ICH seizures were revealed. In patients with increased risk of post-SICH seizures, appropriate surveillance and management of seizures should be carried out.

## Introduction

Spontaneous intracerebral hemorrhage (SICH) is one of the most common critical neurological conditions, with a range of 3.1 to 190 per 100,000 person-years [1–3]. Hemorrhage usually has a great impact on neurological function, and its complications result in a high rate of morbidity and mortality [4–6]. The current management of SICH consists of controlling blood pressure and correcting platelet dysfunction or coagulation defect [7–10]. Surgical evacuation of intracerebral hematoma should be considered in individuals with a large volume of hematoma with a significant mass effect, which surgery providing a survival benefit for patients with superficial intracerebral hemorrhage (ICH) without intraventricular hemorrhage (IVH) rather than ICH that arises in deep locations [11].

A variety of complications can be observed after the onset of SICH [12–14]. Among these complications, a common neurological sequela after hemorrhagic stroke is seizure. Its prevalence is approximately 8.4–19% in adult patients with ICH [15–19]. The appearance of seizures after SICH was found to be associated with a worse outcome, in-hospital complications, such as cardiac arrhythmia or pneumonia, and a higher mortality rate at long-term follow-up [20, 21].

The present study aimed to investigate factors associated with the occurrence of seizures in patients with SICH, and the relationship between post-hemorrhagic seizures and long-term functional outcomes. The authors expected that a number of adverse variables related to the development of the seizure could be prevented or modified to improve the outcome of hemorrhagic stroke treatment.

## Materials and methods

### Patient population

This retrospective study included adult patients with SICH hospitalized at our institute from May 2013 to December 2018. Our medical center is the largest tertiary and university hospital in Thailand and is one of the biggest hospitals in Southeast Asia. Initially, data of patients with intracerebral hemorrhage (ICH) were recovered from the ICD-10 database (code I610-I619). Patients with ICH secondary to trauma, tumor, vascular malformation, cerebral amyloid angiopathy or hemorrhagic transformation after ischemic stroke, patients with a previous history of ICH, cranial surgery, preexisting epilepsy before the onset of ICH, and without complete 2-year follow-up data were excluded from the study. Finally, participants who completed two years of follow-up after the onset of SICH were included for data collection and analysis. The recruited patients were categorized into three groups according to the occurrence of seizures following SICH. These included the group with absence of seizures, early seizures, and late seizures after SICH.

### Seizures after SICH

In patients with the appearance of clinical seizures after stroke, early and late seizures were clarified. The early seizure was defined as the episode(s) of the ictus that clinically presented within 7 days after the hemorrhagic stroke. In contrast, a seizure that developed after a week of the event was defined as a late seizure [22–24].

### Data collection

Demographic, clinical and radiographic characteristics, treatment, hospital complications, and outcome at the follow-up period were collected for data analysis. Demographic data included age, sex, and underlying disease. Duration of symptom before hospitalization, clinical manifestation, and initial level of consciousness assessed by Glasgow Coma Score (GCS) were aggregated as clinical information. In terms of radiographic characteristic on cranial computerized tomography (CT), the collected data focused on the location, lateralization, number and volume of the hematoma, as well as associated intracranial findings caused by the hematoma in the brain, such as brain edema or herniation, shifting of the midline structures, hydrocephalus, visible blood in the ventricle, subdural, or subarachnoid space. Regarding the surgical treatment of hemorrhagic stroke, operative data, including craniotomy with evacuation of hematomas, and temporary or permanent diversion of cerebrospinal fluid (CSF), were cumulated.

Various complications during hospitalization were inspected. They included neurological, cardiovascular, infectious, and renal adverse events. The authors also collected evidence of neurologic deficits as consequences of SICH, radiographic findings on cranial CT, and functional outcome. The latter was evaluated using the Modified Rankin Score (mRS) two years following the onset of hemorrhagic stroke. All data were obtained from the medical records and database of our hospital. Patients who were eventually transferred for long-term treatment to other hospitals were interviewed by telephone with these patients or their caregivers to evaluate the functional outcome.

### Ethical approval

This research was ethically approved by Siriraj Institutional Review Board (SIRB), Faculty of Medicine Siriraj Hospital, Mahidol University, Bangkok, Thailand; Certificate of Approval (COA) number Si 079/2021. Patient data in the study were kept confidential according to the Declaration of Helsinki.

### Consent to participate

Written informed consent to participate was not required for this retrospective study.

### Consent for publication

No personally identifiable data from research participants was revealed in the present article. Written informed consent for publication was not required for this study.

### Statistical analysis

The numerical data were presented as mean, standard deviation, median and range, while the categorical data were displayed as frequency and percentage. Two independent categorical variables were evaluated using Pearson’s Chi-square test or Fisher’s exact test. The mean or median of two independent variables with normal distribution was compared with the independent t-test. For the counterpart with skewed distribution, the Mann-Whitney U test was selected. The Paired Sample T-Test was used to compare the mean or median of two dependent variables. Strength of association was calculated using the odds ratio (OR) and the 95% confidence interval (95% CI). Factors associated with early and late post-SICH seizure were determined by univariate analysis with statistical significance of *p*-value < 0.05. Variables with a *p*-value less than 0.1 obtained from the univariate analysis were further analyzed using multinomial logistic regression. The risk factors of the occurrence of seizures after SICH were investigated using backward selection predictor criteria (Wald) with a probability of removal of 0.10. Statistical analysis was performed using IBM SPSS Statistics 24.0 (The International Business Machine Corporation, IBM Corp., Armonk, NY, USA).

## Results

**Fig. 1:**
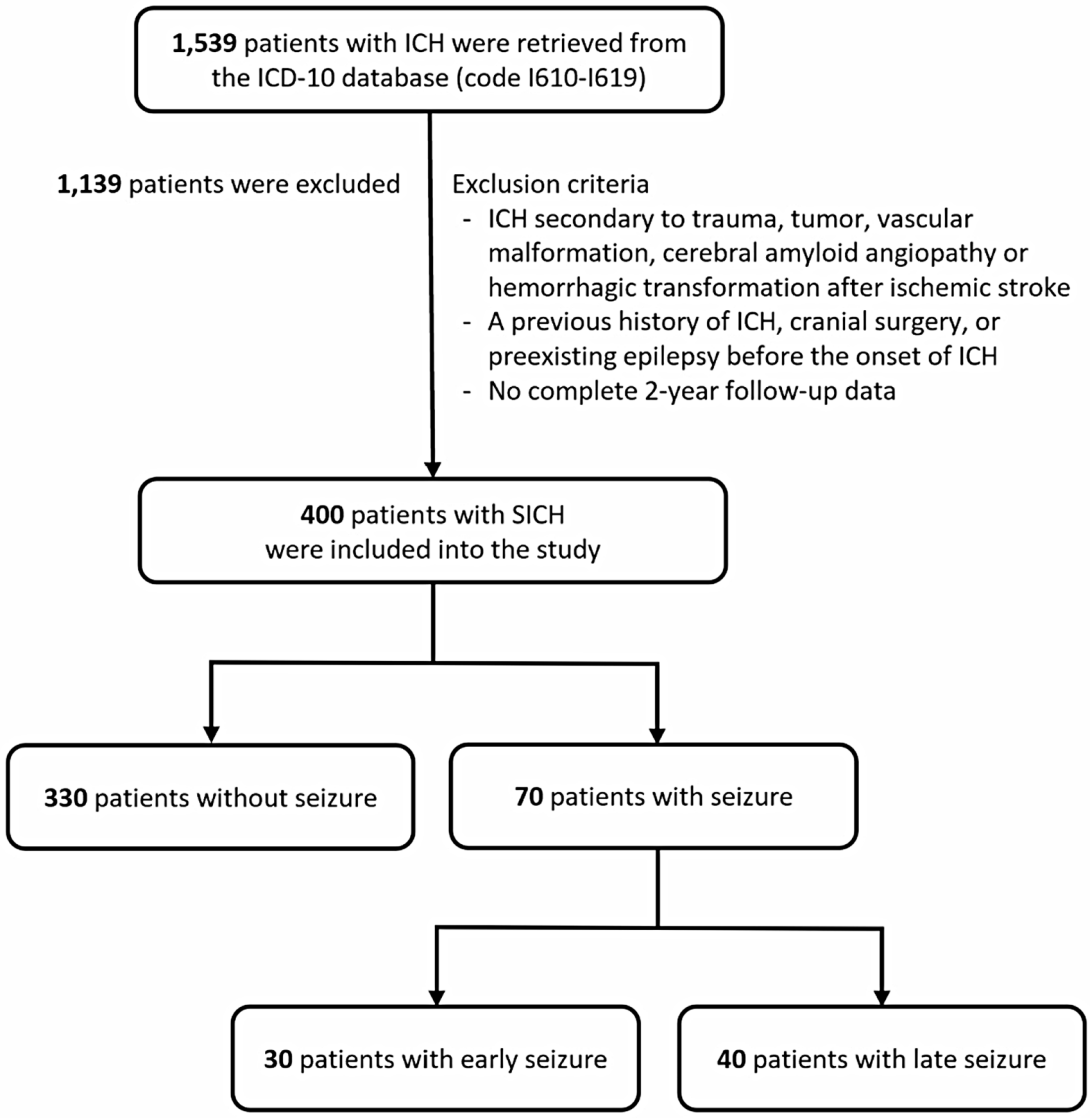
The algorithm of patient selection process

Initially, a total of 1,539 patients were recovered from the ICD-10 database (code I610-I619). Of them, 1,139 patients with the exclusion criteria were excluded. Finally, 400 individuals diagnosed as SICH were enrolled in the study. Of these, 70 (17.5%) patients developed seizure after SICH, divided into early seizure in 30 (7.5%) and late seizure in the remaining 40 (10%) (Fig. 1). There was no patient who had overlapping early and late seizures after SICH. In the univariate analysis of factors associated with the development of early and late seizures after SICH **(**Table 1**)**, deep location, and infratentorial location of ICH were found to be factors with reduction of seizure occurrence. In contrast, low GCS, longer duration of symptoms, presence of neurologic deficit, lobar location of the ICH, higher volume of hematoma, midline shift, brain herniation, subarachnoid hemorrhage (SAH), and subdural hemorrhage (SDH) on initial cranial CT, craniotomy with evacuation of the hematoma were risk factors for seizure development. The strength of association between demographic, clinical, radiographic, and therapeutic variables and the occurrence of seizures after SICH was demonstrated in Table 2. Furthermore, in the univariate analysis of complications during admission and follow-up results **(**Table 3**)**, higher prevalence of nosocomial pneumonia was significantly observed in both of the seizure groups. Similarly, at 2 years of follow-up, the presence of spastic hemiparesis and unfavorable functional outcome defined with mRS of 4 to 6, were significantly associated with the development of seizures following SICH.

**Table 1:** Univariate analysis of factors associated with occurrence of seizure after SICH

				No seizure	Early seizure	Late seizure	*p* value	
				*n* = 330	*n* = 30	*n* = 40		
**Demographic characteristics**								
Age (years), mean ± SD				61 ± 13.5	56.7 ± 14	60.9 ± 11.8	0.239	
Gender, n (%)							0.499	
	Male			206 (62.4)	17 (56.7)	28 (70)		
	Female			124 (37.6)	13 (43.3)	12 (30)		
Underlying disease, n (%)								
	Coronary artery disease			29 (8.8)	2 (6.7)	4 (10)	0.886	
	Chronic kidney disease			25 (7.6)	5 (16.7)	5 (12.5)	0.124	
	Old cerebral infarction			41 (12.4)	3 (10)	5 (12.5)	1.000	
	Atrial fibrillation			14 (4.2)	2 (6.7)	3 (7.5)	0.453	
	Liver disease			11 (3.3)	1 (3.3)	1 (2.5)	1.000	
	Hematologic disease			11 (3.3)	0 (0)	2 (5)	0.533	
	Antiplatelet use			71 (21.5)	6 (20)	9 (22.5)	0.971	
	Anticoagulant use			22 (6.7)	2 (6.7)	5 (12.5)	0.375	
	Thrombocytopenia			9 (2.7)	0 (0)	2 (5)	0.451	
	Coagulopathy			32 (9.7)	2 (6.7)	4 (10)	0.946	
	Alcohol use			123 (37.3)	11 (36.7)	19 (47.5)	0.447	
	Cigarette smoking			85 (25.8)	8 (26.7)	9 (22.5)	0.895	
**Clinical characteristics**								
Duration of symptom (hours), median (range)				3 (2–8)	2 (1–4)	4 (2–10.5)	0.042^a^	
Clinical manifestation, n (%)								
	Neurologic deficit			285 (86.4)	21 (70)	37 (92.5)	0.021^a^	
	Headache			133 (40.3)	14 (46.7)	19 (47.5)	0.572	
	Impaired consciousness			141 (42.7)	16 (53.3)	23 (57.5)	0.132	
	Hemiparesis			277 (83.9)	21 (70)	35 (87.5)	0.110	
	Dysarthria			145 (43.9)	7 (23.3)	18 (45)	0.087	
	Dysphasia			70 (21.2)	3 (10)	7 (17.5)	0.311	
Glasgow Coma Score, median (range)				15 (11–15)	10 (9–15)	13 (9–15)	0.003^a^	
Glasgow Come Score, n (%)							0.005^a^	
		> 12		219 (66.4)	12 (40)	21 (52.5)		
		≤ 12		111 (33.6)	18 (60)	19 (47.5)		
**Radiographic characteristics**								
Lobar location of hematoma, n (%)				58 (17.6)	13 (43.3)	18 (45)	< 0.001^a^	
Volume of hematoma (ml), median (range)				10 (4–26)	25 (13–46)	34 (17–63)	< 0.001^a^	
Associated findings, n (%)								
	Perilesional edema			305 (92.4)	30 (100)	39 (97.5)	0.062	
	Midline shift			155 (47)	18 (60)	33 (82.5)	< 0.001^a^	
	Brain herniation			83 (25.2)	13 (43.3)	23 (57.5)	< 0.001^a^	
	Obstructive hydrocephalus			72 (21.8)	6 (20)	13 (32.5)	0.293	
	Communicating hydrocephalus			19 (5.8)	4 (13.3)	1 (2.5)	0.152	
	Intraventricular hemorrhage			131 (39.7)	12 (40)	16 (40)	1.000	
	Subdural hemorrhage			12 (3.6)	2 (6.7)	5 (12.5)	0.031^a^	
	Subarachnoid hemorrhage			21 (6.4)	7 (23.3)	7 (17.5)	0.001^a^	
**Surgical treatment**								
	Craniotomy with hematoma evacuation			57 (17.3)	13 (43.3)	19 (47.5)	< 0.001^a^	
	Ventriculostomy			51 (15.5)	3 (10)	6 (15)	0.726	
	CSF shunting			16 (4.8)	1 (3.3)	3 (7.5)	0.654	

^a^*p* value < 0.05 indicates statistical significance

CSF, cerebrospinal fluid; n, number; SD, standard deviation

**Table 2:** Strength of association between demographic, clinical, radiographic, and therapeutic variables and the occurrence of seizures after SICH

		OR	(95% CI)	*p* value
**Demographic characteristics**				
	Age	0.99	(0.97–1.01)	0.277
	Male gender	1.08	(0.63–1.85)	0.770
	Coronary artery disease	0.97	(0.39–2.44)	0.954
	Chronic kidney disease	2.03	(0.93–4.45)	0.076
	Old cerebral infarction	0.91	(0.41–2.04)	0.818
	Atrial fibrillation	1.74	(0.60–4.99)	0.306
	Liver disease	0.85	(0.19–3.94)	0.838
	Hematologic disease	0.85	(0.19–3.94)	0.838
	Antiplatelet use	0.99	(0.53–1.87)	0.987
	Anticoagulant use	1.56	(0.64–3.80)	0.332
	Thrombocytopenia	1.05	(0.22–4.96)	0.952
	Coagulation	0.87	(0.35–2.18)	0.771
	Alcohol use	1.26	(0.75–2.13)	0.383
	Cigarette smoking	0.93	(0.51–1.68)	0.798
**Clinical characteristics**				
	Duration of symptom	0.99	(0.98–1.01)	0.390
	Neurologic deficit	0.76	(0.38–1.53)	0.447
	Headache	1.32	(0.79–2.22)	0.292
	Impaired consciousness	1.67	(1.00–2.84)	0.049^a^
	Hemiparesis	0.77	(0.40–1.47)	0.424
	Dysarthria	0.71	(0.42–1.12)	0.207
	Dysphasia	0.62	(0.30–1.27)	0.191
	Glasgow Coma Score ≤ 12	2.18	(1.30–3.68)	0.003^a^
**Radiographic characteristics**				
	Lobar location of hematoma	3.73	(2.15–6.46)	< 0.001^a^
	Volume of hematoma > 10 ml	5.56	(2.82–11.00)	< 0.001^a^
	Perilesional edema	5.66	(0.75–42.46)	0.062
	Midline shift	3.03	(1.72–5.36)	< 0.001^a^
	Brain herniation	3.15	(1.85–5.36)	< 0.001^a^
	Obstructive hydrocephalus	1.34	(0.74–2.40)	0.336
	Communicate hydrocephalus	1.26	(0.45–3.49)	0.658
	Intraventricular hemorrhage	1.01	(0.60–1.72)	0.962
	Subdural hemorrhage	2.94	(1.12–7.77)	0.029^a^
	Subarachnoid hemorrhage	3.68	(1.77–7.66)	0.001^a^
**Surgical treatment**				
	Craniotomy with hematoma evacuation	4.03	(2.33–6.99)	< 0.001^a^
	Ventriculostomy	0.81	(0.38–1.73)	0.581
	CSF shunting	1.19	(0.39–3.67)	0.763

^a^*p* value < 0.05 indicates statistical significance

CI, confidence interval; CSF, cerebrospinal fluid; OR, odds ratio

**Table 3:** Univariate analysis of association between in-hospital complications, outcomes on follow-up, and the occurrence of seizures after SICH

					No seizure	Early seizure	Late seizure	*p* value
					*n* = 330	*n* = 30	*n* = 40	
**In-hospital complications**								
Neurological system, n (%)								
	Delirium				6 (1.8)	1 (3.3)	1 (2.5)	0.827
	Meningitis				11 (3.3)	2 (6.7)	2 (5.0)	0.595
	Myoclonus				2 (0.6)	0 (0)	0 (0)	0.807
	Others				3 (0.9)	0 (0)	0 (0)	0.726
Cardiovascular system, n (%)								
	Heart failure				9 (2.7)	2 (6.7)	0 (0)	0.241
	Atrial fibrillation				4 (1.2)	0 (0)	1 (2.5)	0.641
	Infective endocarditis				1 (0.3)	0 (0)	1 (2.5)	0.164
	Cardiac arrest				2 (0.6)	0 (0)	0 (0)	0.807
Infection, n (%)								
	Pneumonia				76 (23)	14 (46.7)	15 (37.5)	0.004^a^
	Urinary tract infection				40 (12.1)	4 (13.3)	2 (5)	0.431
	Sepsis				8 (2.4)	2 (6.7)	3 (7.5)	0.074
Renal system, n (%)								
	Acute kidney injury				12 (3.6)	1 (3.3)	2 (5)	0.867
	Hyponatremia				48 (14.5)	3 (10)	10 (25)	0.157
Other complications, n (%)					28 (8.5)	0 (0)	3 (7.5)	0.272
**Outcomes on follow-up**								
Neurologic deficit, n (%)								
	Impaired consciousness				44 (13.3)	5 (16.7)	11 (27.5)	0.058
	Hemiparesis				200 (60.6)	19 (63.3)	31 (77.5)	0.113
	Spastic hemiparesis				130 (39.4)	15 (50)	27 (67.5)	0.002^a^
	Dysarthria				48 (14.5)	6 (20)	9 (22.5)	0.343
	Dysphasia				48 (14.5)	6 (20)	9 (22.5)	0.343
Radiographic characteristics, (%)								
	Encephalolamacia				160 (48.5)	12 (40)	26 (65)	0.207
	Resolving hematoma				79 (23.9)	5 (33.3)	7 (17.5)	0.317
	Hydrocephalus				82 (24.8)	7 (23.3)	15 (37.5)	0.411
Functional outcomes at 2 years after SICH								
	mRS, median (range)				2 (1–4)	4 (1–5)	3 (2–5)	0.009^a^
	mRS, n (%)							0.015^a^
		Favorable	mRS 0–3		224 (67.9)	14 (46.7)	21 (52.5)	
		Unfavorable	mRS 4–6		106 (32.1)	16 (53.3)	19 (47.5)	

^a^*p* value < 0.05 indicates statistical significance

mRS, Modified Rankin Score; n, number; SICH, spontaneous intracerebral hemorrhage

All variables in Table 2 with a *p* value less than 0.1 were incorporated in the multivariate analysis **(**Table 4**)**. Factors significantly associated with the appearance of the early seizure included lobar location of hematoma (adjusted OR = 2.68, 95% CI = 1.18–6.08, *p* = 0.018), and GCS ≤ 12 on initial clinical presentation (adjusted OR = 2.92, 95% CI = 1.34–6.39, *p* = 0.007). Lobar location of hematoma (adjusted OR = 3.30, 95% CI = 1.66–6.59, *p* = 0.001), volume of hematoma > 10 ml (adjusted OR = 4.10, 95% CI = 1.42–11.80, *p* = 0.009), and midline shift on initial cranial CT (adjusted OR = 2.53, 95% CI = 1.06–6.03, *p* = 0.036), were found to be factors predicting the occurrence of the late seizure. Finally, risk factors of overall seizure after SICH comprised of lobar location of hematoma (adjusted OR = 3.26, 95% CI = 1.82–5.84, *p* < 0.001), volume of hematoma > 10 ml (adjusted OR = 3.71, 95% CI = 1.78–7.73, *p* < 0.001), and craniotomy with evacuation of hematoma (adjusted OR = 2.32, 95% CI = 1.26–4.26, *p* = 0.007).

**Table 4:** Multivariate analysis of factors associated with the occurrence of seizures after SICH

Variables		Unadjusted OR (95% CI)	*p*-value	Adjusted OR (95% CI)	*p*-value
**Early seizure**					
Lobar location of hematoma					
	Absent	Reference			
	Present	3.73 (2.15–6.46)	< 0.001^a^	2.68 (1.18–6.08)	0.018^a^
GCS on initial clinical presentation					
	> 12	Reference			
	≤ 12	2.18 (1.30–3.68)	0.003^a^	2.92 (1.34–6.39)	0.007^a^
**Late seizure**					
Lobar location of hematoma					
	Absent	Reference			
	Present	3.73 (2.15–6.46)	< 0.001^a^	3.30 (1.66–6.59)	0.001^a^
Volume of hematoma					
	≤ 10 ml	Reference			
	> 10 ml	5.56 (2.82–11.0)	< 0.001^a^	4.10 (1.42–11.80)	0.009^a^
Midline shift on initial cranial CT					
	Absent	Reference			
	Present	3.03 (1.72–5.36)	< 0.001^a^	2.53 (1.06–6.03)	0.036^a^
**Overall seizures**					
Lobar location of hematoma					
	Absent	Reference			
	Present	3.73 (2.15–6.46)	< 0.001^a^	3.26 (1.82–5.84)	< 0.001^a^
Volume of hematoma					
	≤ 10 ml	Reference			
	> 10 ml	5.56 (2.82–11.0)	< 0.001^a^	3.71 (1.78–7.73)	< 0.001^a^
Craniotomy with hematoma evacuation					
	Absent	Reference			
	Present	4.03 (2.33–6.99)	< 0.001^a^	2.32 (1.26–4.26)	0.007^a^

^a^*p* value < 0.05 indicates statistically significance

The following variables with *p* value less than 0.1 in Table 2, were included in the multivariate analysis: underlying disease (chronic kidney disease), clinical characteristics (impaired consciousness, Glasgow Coma Score), radiographic characteristics (lobar location of the hematoma, volume of hematoma > 10 ml, perilesional edema, midline shift, brain herniation, subdural hemorrhage, subarachnoid hemorrhage), surgical treatment (craniotomy with hematoma evacuation)

CI, confidence interval; GCS, Glasgow Coma Score; OR, odds ratio

## Discussion

Seizure is a common neurological consequence after ICH and occurs mainly in the first 72 h after bleeding, especially at the onset of hemorrhagic stroke [25–27]. In general, an early seizure is unlikely to develop into chronic epilepsy; however, the prevalence of epilepsy in patients with SICH is relatively higher than in the general population by age. A rare post-SICH entity is a late-onset seizure. If it occurs, the seizure usually begins within the first 2 years following the onset of bleeding [26]. Pathogenic mechanisms in the development of post-stroke seizure have been proposed. Dysfunction of sodium and calcium channels, disequilibrium between excitatory and inhibitory neurotransmitters, hemosiderin deposition, disruption of the blood-brain barrier, elevated pro-inflammatory cytokines, astrocytic proliferation, structural changes of the brain network and alterations in regional hemodynamics were mentioned as the major processes of epileptogenesis [28–30].

Ion channel dysfunction is a major pathogenic mechanism of post-stroke seizure and epilepsy. When acute brain injury occurs after stroke, functions of sodium and calcium channels are impaired. These ion channel dysfunctions result in abnormal sodium influx and increased depolarization of cell membrane potentials. Then calcium channel is activated, and excessive intracellular calcium influx brings about abnormal neuronal discharges and appearance of seizure [30–35]. Neurotransmitter disequilibrium also involves in development of post-stroke seizure. Increased glutamate release activates neuronal hyperexcitability, whereas protein subunit of receptor and transportation of a main inhibitory neurotransmitter, γ-aminobutyric acid (GABA), are reduced. These mechanisms make vulnerable neurons susceptible to develop hyperexcitability and seizure [28, 30, 36–38]. Leakage of albumin into the brain parenchyma adjacent to hemorrhagic area can induce seizure by stimulation of transforming growth factor (TGF) β on the surface of astrocytes [39]. Deposition of blood substances, including iron and hemosiderin in the brain parenchyma involves in epileptogenesis after hemorrhagic stroke. These substances are highly cytotoxic, and their deposition in the brain tissue leads to inflammation, gliosis, accumulation of hydroxyl radical production and neuronal dysfunction which causes seizure and epilepsy [36, 40, 41]. Mechanisms of post-stroke epileptogenesis as a result of blood-brain barrier (BBB) dysfunction have been proposed. Disruption and dysfunction of BBB can occur in both ischemic and hemorrhage strokes. BBB dysfunction results in extravasation of thrombin, albumin and iron, ion channel imbalances, vasogenic edema, glutamate excitotoxicity, neuroinflammation, and increased TGF-β signaling. These insults can lead to the occurrence of acute symptomatic seizures, unprovoked seizures, and post-stroke epilepsy [30, 42, 43].

Furthermore, several pro-inflammatory cytokines, chemokines, and alarmins play a major role in development of seizure. They include interleukin (IL) 6, IL-1β, tumor necrosis factor (TNF) α, TGF-β, CCL2, CCL3, CCL4, CCL5, CXCL10, CX3CL1, high mobility group box 1 (HMGB1), receptor for advanced glycation end products (RAGE), and toll-like receptor 4 (TLR4). These substances promote neuroinflammation and can induce post-stroke seizure and epilepsy [44]. Glial activation, proliferation, and scarring are associated with epileptogenesis following stroke. These changes interfere ion channel balance, cell membrane depolarization, and neurotransmitter equilibrium, and also affect long-term neuronal function and may contribute to late-onset seizure [25, 33, 45, 46]. Neural structures and networks are always disrupted after stroke. Neurogenesis can occur as a repairing process in the late stage of stroke. Aberrant neurogenesis and maladaptive plasticity may result in neuronal circuit dysfunction and be risky to develop seizure [25, 36, 46, 47]. Finally, alterations in regional cerebral hemodynamics may involve in seizure induction. High perfusion of cerebral blood flow can occur in association with seizure occurrence [48, 49].

Post-stroke seizures have a significant impact on patients in several aspects. The quality of life of patients is impaired, particularly when seizures are not prevented [50]. A study by Misra et al. revealed an association between post-stroke seizure and a higher risk of death, worse functional outcome, a higher risk of disability, and dementia compared to patients without seizure [51]. In terms of mortality, most deaths are caused by existing vascular diseases [50]. However, some studies showed that mortality rate was not associated with post-stroke seizures [52, 53].

The present study investigated the potential factors for the occurrence of seizures following SICH. In the univariate analysis of clinical manifestation, poor initial clinical status determined by the GCS contributed to a risk factor for the early seizure in this study. The literature showed that patients with a disorder of consciousness are at risk of seizures and epilepsy [54, 55]. A large acute hematoma with a significant mass effect causes impaired consciousness and an increased risk of seizures in the acute phase of hemorrhagic stroke. Neurological deficit was significantly found in the late seizure group. The patients with neurologic deficit probably had a hematoma in the lobar region. Over time, after the hematoma has resolved and scar formation occurs, an increased long-term risk of seizure has been reported [36]. Our patients with hemiparesis also had a tendency to develop post-SICH seizure; however, the risk did not reach a statistically significant level. The short duration of the symptoms was found in the early seizure group. Hemorrhagic stroke patients who present with acute symptomatic seizure in the early phase after ICH likely go to the emergency department earlier than those with other presenting symptoms, such as hemiparesis or dysphasia. This could reduce the duration of symptoms in the early seizure group to shorter than in the non-seizure group or the late seizure group.

In our univariate analysis, chronic kidney disease (CKD) was a factor might increase risk of post-hemorrhagic seizure. Nevertheless, its risk did not achieve the statistically significant level (OR = 2.03, 95% CI = 0.93–4.45, *p* = 0.076). Why does CKD increase the risk of post-SICH seizure? Seizure is a neurological consequence found in approximately 10% of CKD patients [56, 57]. Several conditions correlated with CKD can precipitate seizure, such as uremia, electrolyte imbalance, lupus vasculitis, air embolism during hemodialysis, posterior reversible encephalopathy syndrome (PRES), or ischemic or hemorrhagic vascular event [57–59]. Reduction of seizure threshold is a major risk factor of seizure activation in CKD patients, particularly in those with uremia. Approximately one-third of patients with uremic encephalopathy develop seizures [59]. Mechanisms of seizure threshold reduction have been elucidated. The accumulation of uremic toxins in the central nervous system, including blood urea nitrogen, guanidinosuccinic acid, creatinine and creatine, as well as glutamate release, participate in the alteration of the seizure threshold [28, 60–62]. These insults cause neuronal hyperexcitability, neurotoxicity, and seizure stimulation [58–60, 62]. Dialysis and correction of metabolic derangement are helpful for the prevention of recurrent seizures and improving survival outcomes [63, 64]. Furthermore, the incidence of cerebrovascular events in patients with CKD is markedly higher than in patients without CKD up to 5 to 30 times [65, 66]. ICH and SDH are common hemorrhagic events in CKD patients due to coagulopathy, anticoagulant effect, and uremic platelet dysfunction [67, 68]. Following the onset of ICH in patients with CKD, regional cerebral hypoperfusion, decreased transmembrane potential depolarization due to sodium and potassium accumulation, calcium-mediated effects, dysregulation of ICP control, and glutamate release after neuronal death reduce the threshold for seizures and finally induce an episode of seizure [28, 57].

In our radiographic analysis, the superficial location of SICH or lobar hemorrhage was a strong risk factor for the occurrence of seizures. On the contrary, no case with pontine hemorrhage or IVH, and only one with cerebellar hemorrhage developed post-SICH seizure. Furthermore, the early and late seizure groups obviously had a lower proportion of hematoma originating in the putamen and thalamus, compared to the group without seizure. These results indicated that hematoma located in the superficial lobes, especially involving the cerebral cortex, is considered to carry a markedly higher risk of seizure after hemorrhagic stroke compared to hemorrhage in deeper or infratentorial structures, which was consistent with many previous studies [26, 29, 36, 69–74]. From a neurophysiological point of view, electrographic seizures and hyperexcitable patterns (rhythmic delta, periodic discharges or spike wave discharges) on electroencephalography (EEG) were more frequent in the lobar hemorrhage group compared to the counterpart group with deep intraparenchymal hemorrhage [75]. In a previous study of seizures among individuals with lobar hemorrhage, Wiebers et al. found an association between seizures and lobar hemorrhage in the frontal, parietal, or temporal region, but not occipital hematoma [74]. However, our univariate analysis showed relatively higher proportions of prevalence of early and late seizures in patients with lobar hematoma of all cerebral lobes, including the occipital area, compared to those without seizures. Another report by Jung and colleagues demonstrated that hemosiderin deposition in the leptomeningeal and subpial layers of the brain, or the so-called ‘cortical superficial siderosis’, could be observed in a lobar hemorrhagic patient with seizure [76]. This finding could be an epileptogenic factor causing seizure in the lobar hematoma with cortical involvement.

The large volume of ICH was a crucial issue involved in the occurrence of post-hemorrhagic seizures [29]. Acute neuronal injury, mechanical effects of expanded hemorrhage, and irritation of the cerebral cortex due to products of blood metabolism play a major role in the development of early seizures [77], while delayed seizures usually occur as a result of gliotic scars and hemosiderin deposition in the brain caused by a consequence of hemorrhagic stroke [77, 78]. The reaction of astrocytes to adverse brain injury triggers the formation of astroglial scars, leading to seizures and epilepsy [79]. A greater volume of hematoma was a strong risk factor of the development of seizures in both our univariate and multivariate analyzes. In addition to the direct pressure effect of the hematoma on the brain, our evidence of increased ICP, elicited by shift of the midline structures and brain herniation on neuroimaging could participate in the activation of the post-hemorrhagic seizure [80, 81]. In the same way, the progression of cerebral edema was associated with an increased risk of acute seizures [82], but not in the present study.

Bleeding from ICH in the subarachnoid or subdural space was associated with an increased risk of seizure in the univariate analysis. SAH influenced the appearance of the early and late seizure, whereas SDH mainly affected the late seizure. In the same way as with SICH, mechanical compression of the brain surface, cortical irritation from blood products, and cerebral hyperemia result in a reduction in the seizure threshold [83, 84]. In the long term, they can leave cortical scarring and residual brain irritation, leading to delayed seizure or chronic epilepsy [69, 85]. Electrographic seizures are frequently seen on EEG in patients with SAH or SDH [86–88], and prophylactic antiepileptic drugs should be administered in these cases to reduce the burden of seizures [89–91].

Among our surgical interventions for the treatment of SICH, craniotomy with evacuation of hematoma was a solitary risk factor for post-hemorrhagic seizure. This result was consistent with that noted in previous articles [92, 93]. This procedure was performed mainly in patients with large-volume ICH greater than 30 ml and impaired consciousness, which also carried a high risk of seizures. In surgical evacuation of the hematoma, immediate cerebral cortex damage, brain tissue manipulation, retraction edema, followed by hemosiderin deposition and gliotic scar formation, increase the risk of early and late seizures, respectively. A study by Welte et al. reported the benefit of surgical evacuation of large cortical hematoma in reducing the risk of epilepsy in more than 70% of cases [94]. However, their result was not replicable in our study.

In the analysis of the in-hospital event after SICH and the result of treatment, nosocomial pneumonia, spastic hemiparesis at follow-up and unfavorable function outcome (mRS 4–6) at 2 years after SICH, were parameters that increased the risk of seizures. During the acute phase of hemorrhagic stroke, patients tended to be bedbound concurrently with the presence of potential factors of seizure, such as major neurologic dysfunction and decreased level of consciousness. Due to impaired consciousness, they were prone to develop aspiration pneumonia during hospital stay. Additionally, nosocomial aspiration pneumonia was associated with a poor outcome and 90-day mortality after discharge from hospital [95]. Spastic hemiparesis could be a physical remnant of SICH and gliosis arising in the cortical and subcortical regions involving the pyramidal pathway. Gliotic scarring in this specific area is likely to be vulnerable to epileptogenesis. Our patients who showed poor functional outcome two years after SICH also had a higher risk of occurrence of seizures. The number of studies that mentioned the association between functional outcome and risk of post-ICH seizure was rather scarce. Only two studies and the present research revealed this association. Delayed seizure was related to a worse functional outcome, but this relationship was removed after adjustment of genetic markers and neuroimaging of small vessel disease of the brain [96]. Samake et al. showed a negative impact of late seizure on functional outcome and rehabilitation. Stroke individuals with a late seizure had a poorer neurological functional recovery [97].

Regarding our multivariate analysis, lobar location of hematoma was a solitary factor associated with the early, late, and overall seizures. Volume of hematoma larger than 10 ml was also a factor associated with both late and overall seizures. Both of them were similar to factors used in the CAVE score to predict late seizures after ICH [98]. Regarding research studies since 2000, the prevalence of posthemorrhagic seizure ranged from 4 to 41.8% [99], while ours was 17.5%. Among research articles that focused on risk factors for post-ICH seizures, factors were classified mainly as early and late seizures. Early seizure was shown to be strongly related to cortical involvement of ICH [19, 100, 101], while the late counterpart was also associated with cortical involvement and additional factors, including elevated international normalized ratio (INR) level, subdural hematoma, and other neurologic conditions resulting in astrogliosis, such as prior lobar ICH, dementia, or white matter disease [96, 99]. These findings corresponded to the paradigm of posttraumatic epilepsy. Cellular biochemical dysfunction was the consequence of early seizures, while gliosis and the development of the meningocerebral cicatrix were the main pathophysiology of late seizures [102].

The literature review of the factors associated with the occurrence of seizures following SICH is shown in Table 5 [15–20, 75, 96, 98–101, 103–122]. In regards to key findings of the studies, lobar hemorrhage and cortical involvement of ICH were the most common risk factors encountered in the majority of studies, including the present study [15–17, 19, 20, 75, 96, 98, 100, 101, 103–106, 108, 110–113, 115–120]. Large volume of hematoma was an independent factor associated with the occurrence of post-hemorrhagic seizure [16, 98, 99, 106, 111, 115, 120]. Our study also revealed that ICH volume more than 10 ml was associated with late and overall seizures. Conversely, only a study of Passero et al. reported small ICH volume as a risk factor of immediate seizure after hemorrhagic stroke [100]. Younger age was a common risk factor associated with the appearance of seizure in many studies [15, 19, 20, 98, 111, 113, 115, 116, 119, 120, 122], but not in our study. We did not find statistically significant correlation between patient age and seizure development. On the other hand, some researches showed that patients older than 65 years had a greater risk of post-hemorrhagic seizure [106, 110].

**Table 5:** The literature review of factors associated with occurrence of seizure after ICH [15–20, 75, 96, 98–101, 103–122]

Authors (year) [reference]	Study design	*n*	Length of follow-up	Prevalence of seizure (%)	Factors associated with occurrence of seizure after SICH		
					Early seizure	Late seizure	Overall seizures
Bladin et al. (2000) [104]	Prospective	265	9 m	10.6	-	-	Cortical involvement
Passero et al. (2002) [100]	Retrospective	761	30 d	8.1	Immediate seizure^*^: lobar hemorrhage, small ICH volume Early seizure: lobar hemorrhage, neurologic complications Protective factor: prophylactic AEDs	-	-
Yu et al. (2002) [121]	Retrospective	75	2 y	25.3	-	Temporal ICH	Alcohol use
De Reuck et al. (2007) [108]	Retrospective	14	-	Early 42.9; late 57.1	-	-	Lobar hemorrhage, frontal lobe involvement
Yang et al. (2009) [120]	Retrospective	243	3 y	8.2	-	-	Cortical involvement, large ICH volume, young age
Garrett et al. (2009) [99]	Retrospective	110	15 m	41.8	ICH volume, SAH	Elevated INR on admission, SDH	-
Beghi et al. (2011) [103]	Prospective	105	7 d	16.2	Risk factor: cortical involvement Protective factor: hyperlipidemia	-	-
De Herdt et al. (2011) [15]	Prospective	522	6 m	13.6	Cortical involvement	Previous ICH, cortical involvement, young age, severe neurologic deficit at admission	-
Woo et al. (2012) [19]	Retrospective	263	19.5 m	8.4	Cortical involvement, young age	Cortical involvement, communicating hydrocephalus	-
Rossi et al. (2013) [117]	Prospective	325	2.2 y	9.5	-	-	Cortical involvement, lobar brain microbleeds
Qian et al. (2014) [116]	Not mentioned	935	3 m	13.9	Immediate seizure^*^: low GCS on admission, subcortical location of ICH, young age Early seizure: subcortical location of ICH	Subcortical location of ICH, young age, surgical evacuation of hematoma	-
Madžar et al. (2014) [17]	Retrospective	464	1 y	10.7	-	-	Lobar hemorrhage, sepsis, alcohol use
Guth et al. (2014) [109]	Prospective	234	3 m	4	Subarachnoid extension of ICH	-	-
Haapaniemi et al. (2014) [98]	Retrospective	993	2.7 y	9.2	-	Cortical involvement, age < 65 y, ICH volume > 10 ml, early seizure	
Li et al. (2015) [20]	Prospective	3,216	12 m	4.3	-	-	Age < 60 y, AF, stroke severity (GCS ≤ 8), lobar hemorrhage, pneumonia
de Greef et al. (2015) [107]	Retrospective	857	> 193 d	9.8	-	Early seizure	-
Neshige et al. (2015) [115]	Retrospective	1,920	61 m	6.6	-	-	Risk factors of seizure: cortical involvement, non-hypertensive ICH, young age, severe neurological deficits evaluated by NIHSS Risk factor of recurrent seizure: large ICH volume
Biffi et al. (2016) [96]	Retrospective	872	3.9 y	10	-	Dementia, multiple previous lobar hemorrhages, cortical involvement, white matter disease on neuroimaging, exclusively lobar microbleed	-
Brüning et al. (2016) [105]	Prospective	484	7 d	10.7	Cortical involvement	-	-
Lahti et al. (2017) [112]	Retrospective	615	6.4 y	13.5	-	-	Risk factors: subcortical location of ICH Protective factor: hypertension
Mehta et al. (2018) [18]	Retrospective	220,075	-	11.9	-	-	Risk factors: high categorical van Walraven score, encephalopathy, alcohol use, solid tumor, previous stroke Protective factors: old age, uncomplicated DM, renal failure, fluid and electrolyte disorders
Liao et al. (2019) [114]	Prospective	297	7 d	3	High NIHSS on admission, coronary artery disease	-	-
Kwon et al. (2020) [111]	Prospective	2,507	12 m	3.1	-	Young age, use of AEDs, large ICH volume, lobar hemorrhage, surgical evacuation of hematoma	-
Sheikh et al. (2020) [75]	Retrospective	128	-	22	-	-	Risk factor for electrographic seizure: lateralized rhythmic delta activity, lobar hemorrhage
Zöllner et al. (2020) [122]	Retrospective	19,331	-	4	-	-	Infection, GCS < 13, previous stroke, young age
Law et al. (2020) [113]	Prospective	2,325	90 d	8.3	Young age, previous stroke, lobar hemorrhage, high NIHSS on admission	-	-
Sarfo et al. (2021) [118]	Not mentioned	897	-	17.7	-	-	Lobar hemorrhage, alcohol use
Wong et al. (2021) [101]	Retrospective	177	3 y	13.6	Cortical involvement	Cortical involvement	-
Wang et al. (2021) [119]	Retrospective	602	24 m	7.8	-	Lobar hemorrhage, age < 65 y, NIHSS *≥* 15, early seizure	-
Bunney et al. (2022) [106]	Retrospective	864	7 d	8.7	Cortical involvement, age > 65 y, ICH volume > 10 ml	-	-
Guo et al. (2022) [16]	Meta-analysis	32,162	7 d– 7.2 y	9.5	-	-	Risk factors: cortical involvement, lobar hemorrhage, large ICH volume
Huang et al. (2023) [110]	Retrospective	408	4 y	12.7	-	Male, age > 65 y, cortical involvement, early seizure	-
The present study	Retrospective	400	2 y	17.5	Lobar hemorrhage, GCS ≤ 12	Lobar hemorrhage, ICH volume > 10 ml, midline shift on cranial CT	Lobar hemorrhage, ICH volume > 10 ml, craniotomy with evacuation of hematoma

^*^Immediate seizure is defined as an occurrence of seizure within 24 h following the onset of spontaneous intracerebral hemorrhage

-, not reported; AEDs, antiepileptic drugs; AF, atrial fibrillation; d, day; CT, computerized tomography; DM, diabetes mellitus; GCS, Glasgow Coma Score; ICH, intracerebral hemorrhage; INR, international normalized ratio; m, month; n, number of cases; NIHSS, The National Institutes of Health Stroke Scale; SAH, subarachnoid hemorrhage; SDH, subdural hemorrhage; SICH, spontaneous intracerebral hemorrhage; y, year

Another common risk factor of post-ICH seizure was stroke severity assessed by GCS or The National Institutes of Health Stroke Scale (NIHSS). A large number of studies, including our study reported a significantly higher chance of seizure occurrence in patients with lower GCS or higher NIHSS score [15, 20, 113–116, 119, 122]. Interestingly, some studies revealed that patients who had early seizures after SICH carried a higher risk of subsequent late seizure [98, 107, 110, 119]. However, we did not find this tendency. In the present study, none of our patients who had early seizure developed late seizure after ICH. Previous stroke was a risk factor of post-ICH seizure in some studies [15, 18, 96, 113, 122]. Our study excluded patients with previous stroke and other insults to eliminate bias and confounding variables in statistical analysis.

In contrast to the risk factors, several variables have been advocated as protective factors of post-ICH seizure, such as prophylactic antiepileptic drugs, old age, uncomplicated diabetes mellitus [18, 100, 103, 112]. One sensible factor which may reduce risk of post-ICH seizure was surgical evacuation of hematoma. Welte and colleagues showed the benefit of surgical evacuation of large cortical hematoma in reduction of the risk of epilepsy in more than 70% of their patients [94]. However, this result was not found in our and previous studies. On the contrary, ours and a study of Kwon et al. [111] proposed that surgical evacuation of hematoma was a risk factor of seizure development following SICH. Furthermore, we did not find any variable which could be the protective factor of the seizure.

Regarding strength of our study, we investigated risk factors of early, late, and overall seizures after hemorrhagic stroke, while all of previous studies reported either early or late seizures, early and late seizures, or only overall seizure. Additionally, we totally excluded patients with other types of brain insult, cranial surgery, or preexisting epilepsy before the onset of SICH to diminish bias and confounding factors in statistical analysis. On the contrary, our limitation was lack of analysis of immediate seizure within 24 h following the hemorrhagic stroke onset. The literature review reported that lobar hemorrhage, small ICH volume, low GCS on admission, subcortical location of ICH, and young age were risk factors of the immediate seizure [100, 116]. In summary, based on our results, antiepileptic drugs should be prescribed in hemorrhagic stroke patients with lobar hemorrhage, large size of hematoma, impaired consciousness, who have midline shift on cranial CT, or who undergo craniotomy and evacuation of hematoma.

In addition to these analyzed risk factors for post-ICH seizure, some biomarkers were investigated as predictors of seizure occurrence. Genetic and molecular patterns were hypothesized to have an effect on the appearance of seizure. Regarding genetic biomarkers, the frequency of T allele and soluble CD40 ligand (sCD40L) level, CC genotype of transient receptor potential cation channel subfamily M member 6 (TRPM6), and rs671 A allele were significantly higher in patients with post-stroke epilepsy than those in the control groups [123–125]. Downregulations of miR-4325 and miR-4317 were identified as candidate biomarkers for post-ICH late seizure [126]. Pro-inflammatory cytokines activate neuroinflammatory cascade in stroke patients. IL-6 and IL-1β overexpression, high endostatin levels, and decreased S100 calcium-binding protein (S100B), heat shock protein 70 kDa (Hsp70), and neuropeptide Y (NPY) levels in the blood were correlated with post-stroke epilepsy [127–129]. S100B and Hsp70 involves in maintenance of BBB integrity. Decreased levels of S100B and Hsp70 may result in disruption of BBB and seizure [130, 131]. The low serum level of Hsp70 can be utilized in prediction of post-stroke epilepsy [127, 132]. During the first 6 h after stroke onset, high neural cell adhesion molecule (NCAM) and low tumor necrosis factor receptor 1 (TNFR-1) blood levels were related with acute symptomatic seizures [133]. A study conducted by Eriksson et al. revealed that tau protein, glial fibrillary acidic protein (GFAP), neurofilament light (NFL), S100B, and neuron-specific enolase (NSE) had high sensitivity and specificity for post-stroke epilepsy [134]. Abraira and coworkers found that many types of protein involved in epileptogenesis after stroke. In analysis of validation, tumor necrosis factor superfamily 14 (TNFSF-14) was a solitary biomarker having statistically significant downregulation in patients with post-stroke epilepsy compared with control group. This biomarker may serve as a predictor for the development of post-stroke seizure [135]. Furthermore, IL-6 was found to be a biomarker independently associated with recurrent seizure in patients who had first episode of post-stroke seizure. IL-6 mRNA expression level in patients with recurrent seizure was significantly higher than that in control group [136]. Although several genetic and molecular biomarkers have been proposed to be predictors of post-stroke seizure and epilepsy, no single biomarker can be used in the present clinical practice. They require further studies and validations for clinical application.

Some limitations of this study should be clarified. The study design was retrospective in style. A number of uncontrollable variables should be noted. First, in some patients, the evidence of subtle or electrographic seizure might be lost. Seizure occurrences after SICH were clinically evaluated from interviews at follow-up visits without routine confirmation by EEG. Second, the timing of the seizure related to the onset of stroke is an important factor in determining the risk of epilepsy [36]; however, we only classified the seizure into early and late, and did not investigate the actual duration of time between the onset of the stroke and the first episode of the seizure. Third, the results of the present study were exclusively obtained from the database of our hospital which was a large tertiary medical center with the number of beds more than 2,200 beds. Therefore, the generalizability of our results may be limited for use in smaller hospitals or other hospitals with different patient characteristics or different contexts. However, the final results obtained from this study were logical and most of ours were compatible with results of previous studies. Fourth, in statistical point of view, results obtained from regression model may have diminished generalizability, and may be limited in defining confounders and explanatory variables of interest. Furthermore, because the current study was a retrospective cohort instead of a survival analysis, biases and confounding problems might incur. However, the authors tried to reduce them by recruitment of all consecutive cases during the study period, and careful determination of inclusion and exclusion criteria.

## Conclusions

ICH in lobar location, volume of hematoma > 10 ml, GCS ≤ 12 on initial clinical presentation, midline shift on initial cranial CT, and craniotomy with evacuation of hematoma were identified as risk factors of seizure after SICH. Additionally, the occurrence of seizures was associated with poor functional outcome two years after the onset of ICH.

## Data Availability

No datasets were generated or analysed during the current study.
